# Holmes Tremor in CADASIL Responsive to Multi-Targeting Deep Brain Stimulation (DBS): An Educational Case with Video and Electrophysiology

**DOI:** 10.5334/tohm.1128

**Published:** 2026-05-08

**Authors:** Fahimeh H. Akhoundi, Katherine Longardner, Dietrich Haubenberger, Robert Hess, Brenton A. Wright, Sharona Ben-Haim

**Affiliations:** 1 Department of Neurosciences, Parkinson and Other Movement Disorders Center, University of California San Diego, CA, USA; 2 Department of Neurological Surgery, University of California San Diego, California, USA

**Keywords:** Holmes tremor, Cerebral Autosomal Dominant Arteriopathy with Subcortical Infarcts and Leukoencephalopathy (CADASIL), NOTCH3, Deep Brain Stimulation (DBS), Electrophysiology, Tremor

## Abstract

**Background:**

Holmes tremor (HT) is a disabling condition, often resistant to medical therapy.

**Case report:**

A 51-year-old woman with cerebral autosomal dominant arteriopathy with subcortical infarcts and leukoencephalopathy (CADASIL) presented with severe right upper limb HT unresponsive to multiple medications. She underwent deep brain stimulation (DBS) with dual-lead, triple-targeting of the left ventral intermediate nucleus (VIM), posterior subthalamic area (PSA), and globus pallidus interna (GPi) with improvement in tremor.

**Discussion:**

To our knowledge, this case represents the first reported instance of HT in CADASIL and highlights the potential of multi-target DBS for complex, treatment-refractory hyperkinetic movement disorders.

**Highlights:**

We describe the first reported instance of Holmes tremor occurring as a manifestation of CADASIL, successfully treated with triple-target deep brain stimulation of the VIM, PSA, and GPi. This case demonstrates the potential of multi-target DBS to modulate complex motor networks in treatment-resistant tremor and enhance functional outcomes.

## Introduction

Holmes tremor (HT) is a high-amplitude, low-frequency (<5 Hz) tremor affecting distal and proximal upper limb muscles, typically presenting asymmetrically, or unilaterally when arising from a unilateral lesion. It appears at rest and worsens with posture and movement, often causing significant disability [1]. Also called rubral, midbrain, or thalamic tremor, it was first described by Benedikt in 1889 [2], and later defined by Holmes in 1904 [3]. HT is usually due to structural lesions affecting either the brainstem, thalamus, or cerebellum, and arises from dysfunction in motor control networks, including the nigrostriatal, dentato-rubro-thalamic, and cerebello-thalamo-cortical pathways [2]. Maladaptive rhythm generation and aberrant synaptic regeneration after injury likely underlie its pathophysiology, supported by delayed onset of HT after a brain lesion, which may range from weeks to years [4].

Two subtypes of HT have been described. Thalamic HT features slow, large-amplitude, proximal tremor with dystonia, distal choreoathetoid movements, and proprioceptive loss. Midbrain HT manifests with myorhythmic rest tremor, large amplitude proximal tremor, and mild distal dystonia [5]. Other accompanying clinical features may include ataxia, hemiparesis, and oculomotor abnormalities [4].

Etiologies producing HT include strokes, demyelination, trauma, and rarely, infections and tumors [6]. While various oral medications have been tried [7,8], HT is often resistant to pharmacological therapy. The most effective symptomatic relief has been achieved through neurosurgical intervention such as lesioning procedures or deep brain stimulation (DBS) [9]. Since 2001, reports have shown that DBS is effective for HT [10], usually targeting the ventral intermediate nucleus (VIM) of the thalamus and globus pallidus interna (GPi) [11]. Dual targeting, i.e., where electrodes are implanted in two distinct brain regions, either via separate leads or a single electrode traversing multiple targets, has also been employed.

We report a patient with HT and thalamic pain syndrome due to cerebral autosomal dominant arteriopathy with subcortical infarcts and leukoencephalopathy (CADASIL) who underwent DBS with triple targeting of the VIM, posterior subthalamic area (PSA) and GPi, with accompanying electrodiagnostic testing and video recordings pre- and post-surgery. This case illustrates the efficacy of multi-target DBS in treatment-resistant HT.

## Case Presentation

**Figure 1:**
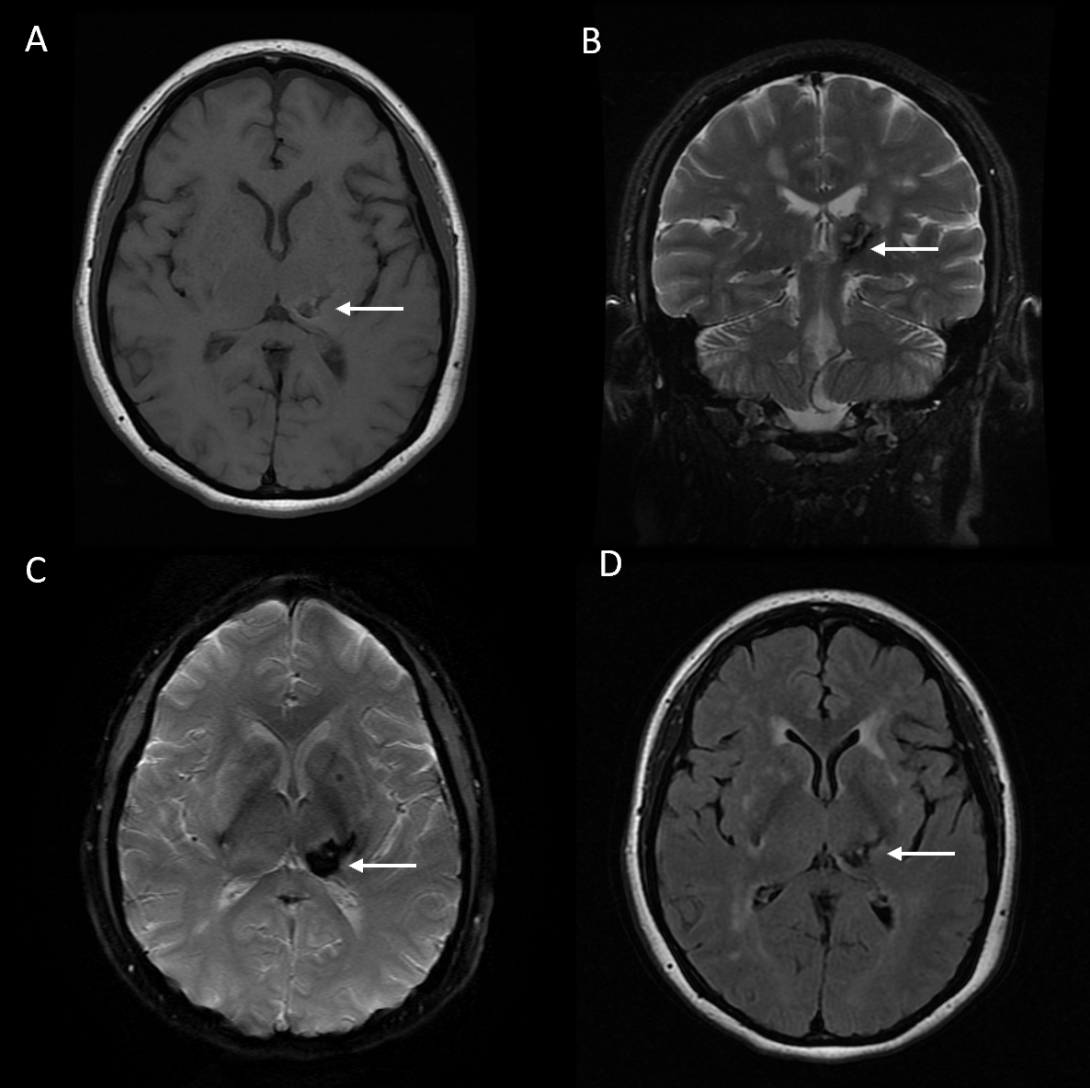
Axial brain magnetic resonance imaging (MRI) T1 **(A)**, coronal T2 **(B)**, axial susceptibility-weighted imaging (SWI) **(C)** and axial fluid-attenuated inversion recovery (FLAIR) **(D)** sequences at the time of the patient’s second stroke, showing acute intracerebral hemorrhage in the left thalamus (white arrow), accompanied by smaller foci of microhemorrhages in left globus pallidus and right inferior putamen. Multiple signal changes in the external capsule, periventricular areas, and centrum semiovale are also observed.

A 51-year-old right-handed woman was referred for intractable right upper limb movements. Her symptoms began four years earlier with right hemibody hypoesthesia and pain, abnormal right arm and leg posturing, and disabling right hand tremor. Her history was notable for hypertension, diabetes, and multiple strokes. Her first stroke occurred at age 43 years, manifesting with right visual hemifield defects and right-hand posturing and numbness. MRI revealed acute left subcortical infarcts and multiple chronic-appearing infarcts. She started taking aspirin daily. At age 45, she experienced acute right-sided numbness, weakness, and short-term memory impairment, which improved spontaneously within two days. MRI demonstrated an acute left thalamic hemorrhage, with small older hemorrhagic foci in the basal ganglia and left subcortical white matter hyperintensities (Figure 1). At age 47, she developed gradual onset, right-sided symptoms including progressively worsening arm and leg dystonic posturing, large amplitude tremulous upper limb movements, and central pain syndrome.

Medications included metformin, atenolol, valsartan, aspirin, and atorvastatin. Family history was notable for recurrent transient ischemic attacks in her mother and multiple strokes and dementia in her maternal grandmother.

Initial neurologic examination demonstrated mild cognitive impairment characterized by deficits in delayed recall with preserved encoding and reduced letter fluency. Cranial nerve findings included mild dysarthria, right hemifacial hypoesthesia, and mild right lower facial weakness. Motor examination revealed slightly reduced strength throughout the right limbs, with right-sided hyperreflexia. She exhibited a right upper extremity tremor involving both proximal and distal muscles, with amplitude <3 cm at rest, increasing to 3–10 cm during posture, and exceeding 10 cm during action. The tremor showed postural, kinetic, and intention properties, fulfilling all components of action tremor. Additional findings included right-sided hemidystonia, dysmetria more pronounced in the upper limb (Video 1), and hemiface and hemibody allodynia with reduced sensation to pain and temperature. Proprioception was normal. She had a hemispastic, hemidystonic gait but could walk independently.

**Video 1:** *Pre-DBS evaluation* demonstrates dystonic posturing of the right upper limb with predominant distal limb involvement, with superimposed mild rest tremor. Tremor emerges during posture and increases with action and intention, accompanied by marked dysmetria of the right upper limb. *Post-DBS evaluation* shows a significant reduction of tremor in the DBS-ON state, particularly during posture, with no appreciable improvement in dysmetria. A longer version of the examination, including gait assessment, is available as Supplementary Video online.

**Figure 2:**
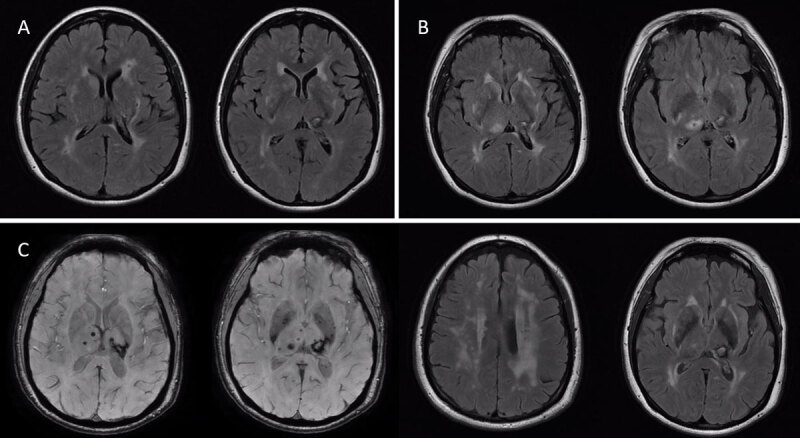
Axial brain MRIs performed at ages 46 **(A)**, 47 **(B)**, and 49 **(C)** years demonstrating the evolution of neuroimaging findings over time, including hemorrhagic subcortical lesions, especially in the left thalamus, and subcortical white matter lesions (A and B, axial FLAIR sequences and C, axial SWI and FLAIR sequences).

Brain MRI at age 47 showed marked bilateral symmetrical periventricular T2 white matter hyperintensities involving the basal ganglia and external capsules bilaterally (Figure 2). Multiple microhemorrhages were evident in the basal ganglia, pons, and cerebellum, along with chronic thalamic strokes. Extensive evaluation for recurrent strokes, including coagulopathy and vasculitis serologies, methylenetetrahydrofolate reductase (*MTHFR*) gene testing, transcranial Doppler with microembolic detection, and magnetic resonance angiography, was unrevealing. Given her neuroimaging findings and family history, genetic testing was performed, revealing a heterozygous variant of uncertain significance (VUS) in NOTCH3: c.2149C>T (p.Arg717Cys). Skin biopsy showed characteristic granular osmiophilic material in the tunica media of the dermal arterioles, establishing pathogenicity and the diagnosis of CADASIL. This variant has since been classified as ‘likely pathogenic’ and has been associated with CADASIL in previous reports [12,13,14].

She was trialed with multiple medications for symptomatic treatment of HT, including carbidopa/levodopa, clonazepam, and onabotulinum toxin A injected in her right arm muscles – all without benefit. Her persistent, disabling tremor of the dominant arm, compounded by severe thalamic pain syndrome, prompted consideration of DBS.

## Clinical neurophysiology studies before DBS

**Figure 3:**
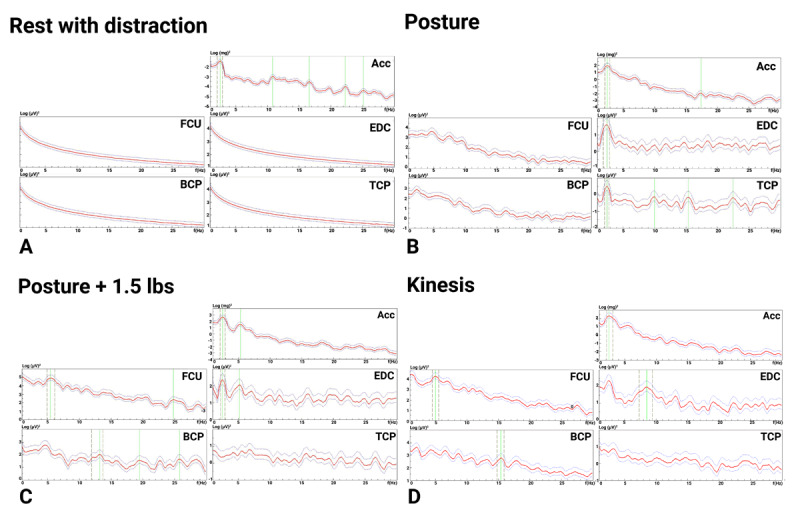
**Electromyography and accelerometry recording from the right upper limb prior to deep brain stimulation surgery**. Each figure depicts a power spectrum, with the x-axis displaying tremor frequency, and the y-axis depicting tremor power, which is the standard measure of tremor amplitude when quantified using accelerometers. At rest during distraction maneuvers (serial seven subtraction), there is a 2.5 Hz right hand tremor detected by the accelerometer **(A)**. During posture with the arm extended, there is a large amplitude 2.5 Hz tremor with a wide peak involving the proximal arm muscles (biceps and triceps) **(B)**. With the addition of 1.5 lbs. to the right hand, the postural tremor frequency remains unchanged (2.5 Hz) **(C)**. There is also a 2.5 Hz right hand kinetic tremor **(D)**. This 2.5 Hz tremor primarily involves the proximal muscles, is present at rest and during posture and kinetic maneuvers, and does not change with weight-loading, consistent with Holmes tremor. Abbreviations: Acc: accelerometer, BCP: biceps EDC: extensor digitorum communis; FCU: flexor carpi ulnaris; TCP: triceps.

Given the patient’s mixed hyperkinetic disorder with right-sided ataxia, dystonia, and HT, electrodiagnostic studies for tremor were performed on her right upper limb as previously described [15,16] to objectively assess the tremor characteristics and to facilitate differential diagnosis and phenotyping. In summary, four-channel non-invasive surface electromyography (EMG) polygraphy was used with electrodes placed on the wrist flexors and extensors, biceps, and triceps muscles along with an accelerometer placed over the dorsum of the right hand (Kistler, sensitivity 20 mV/g), recorded uniaxially in the z-axis, perpendicular to the ground. Electrodiagnostic testing showed a rhythmic, reproducible, and central ~2.5 Hz tremor in the right upper limb primarily involving the proximal muscles, present at rest during distraction maneuvers and during posture and kinetic maneuvers, that did not change with weight-loading, consistent with HT (Figure 3).

After undergoing multidisciplinary pre-DBS evaluation, she was deemed a good candidate and she decided to proceed. Two segmented, directional, wide DBS leads (Abbott Infinity™ system) were placed: one in the left thalamic-subthalamic area (accessing the VIM proximally and posterior subthalamic area (PSA) and zona incerta distally) and another in the left globus pallidus interna (GPi) (Supplemental Figure 1). With respect to surgical technique, although GPi and VIM electrodes have previously been placed through a single burr hole, in this case two separate burr holes within a single scalp incision were used to allow an optimal posterior trajectory for the VIM/PSA–ZI lead while maintaining safe stereotactic angles. Immediately after surgery, the patient experienced a microlesion effect with decreased RUE tremor amplitude; she was able to feed herself with a fork that night for dinner for the first time since her tremor began. The microlesion effect was temporary, and tremor returned to its preexisting level of severity until DBS was activated. Intraoperative stimulation of each target independently resulted in clear tremor improvement, and postoperative monopolar reviews were performed separately for the VIM/PSA and GPi leads. However, prolonged single-lead activation was not pursued in the chronic postoperative setting, and both leads were activated concurrently to leverage potential synergistic effects across cerebellothalamic and basal ganglia motor circuits. After eight weeks of DBS programming, the following parameters were most effective for tremor control: monopolar setting, Case + 2–, 2.5 V, 60 μs and 130 Hz for the VIM/PSA electrode and Case + 12–, 2 V, 60 µs and 130 Hz for the GPi electrode. The patient was specifically monitored for stimulation-related side effects, including gait imbalance and ataxia, particularly during VIM/PSA stimulation. PSA stimulation via contact 1 was associated with transient sensory symptoms, including right arm tightness, and dizziness. Although baseline gait impairment was present, the patient remained independently ambulatory, and no clinically meaningful worsening of gait, balance, or ataxia was observed with the selected chronic stimulation settings.

Tremor was quantified with the Fahn-Tolosa-Marin tremor rating scale (FTMTRS) [17], pre-DBS and 24 months after DBS. We report Part A limb subscores (each domain—rest, posture, action/intention—rated 0–4; limb range 0–12). Pre DBS, the right upper extremity subscore was 8 (rest 1, posture 3, action/intention 4) in the right upper limb and 3 (posture 1, action/intention 2) in the right lower limb. Post-DBS with stimulation off, the right upper extremity subscore was 8 (rest 1, posture 3, action/intention 4), and improved to 6 (rest 0, posture 3, action/intention 3) with DBS on. The right lower extremity improved from 3 (posture 1, action/intention 2) to 1 (posture 0, action/intention 1). Overall, there was a 36.4% reduction from baseline in the right limbs subscore. Since only the tremor in the affected limbs was the focus of this evaluation, only Part A limb subscores are reported. Video 1 shows her exam 24 months post-DBS, in the off and on states. DBS did not alleviate her thalamic pain syndrome, and despite improvement of the tremor, her right upper limb motor control remained impaired due to dysmetria. Given the marked temporal variability that is characteristic of HT, functional outcomes beyond rating scales are particularly informative; in this case, the patient demonstrated clinically meaningful improvement, including restoration of self-feeding with a fork and improved use of the right hand in daily activities, and she was pleased with the results. It is worth noting that the only medication the patient was receiving for her abnormal movements was clonazepam (2 mg twice daily) prior to DBS, which was reduced to 1 mg twice daily after stimulation was turned on.

Clinical neurophysiology repeated with the DBS off and on showed attenuation of tremor with the DBS on (Supplemental Figure 2). Interestingly, and consistent with prior reports [18], DBS-related artifacts in surface EMG recordings were minimal when recordings were obtained from distal upper limb muscles with a ground electrode placed near the muscle of interest.

## Discussion

We report a 51-year-old woman with a CADASIL-related left thalamic stroke who later developed right upper limb HT and right hemibody pain syndrome that were unresponsive to pharmacological therapy. She underwent DBS triple-targeting the left VIM, PSA, and GPi with two electrodes implanted simultaneously, with improvement of her tremor amplitude and functional outcome. This case illustrates the role of multi-target DBS in treating refractory HT and, to our knowledge, represents the first genetically-confirmed CADASIL patient reported with HT.

HT is a lesional tremor, and multiple motor control networks are involved. Motor phenomenology is thought to arise from combined dysfunction of nigrostriatal, cerebellothalamic, and dentatorubral pathways, which accounts for the frequent coexistence of other movement disorders, such as dystonia, dysmetria, parkinsonism, and choreoathetosis. Additional neurological signs, including ophthalmoplegia or spasticity, may occur when lesions extend into adjacent structures. In our patient, the rest tremor may not necessarily reflect nigrostriatal dysfunction since it did not appear to follow a dopaminergic pattern, whereas the action components (postural, kinetic, and intention) were attributable to dentatorubral and cerebellothalamic involvement and were non-dopaminergic. Notably, her tremor amplitude improved more than dysmetria/ataxia following DBS. Given the multifaceted pathophysiology of her tremor and the coexisting dystonia, we anticipated that stimulation of a single target would be insufficient to provide adequate symptom control. Therefore, a multi-target approach was selected to modulate the distinct neural circuits contributing to her symptoms: GPi to influence basal ganglia/nigrostriatal pathways, and VIM/PSA to modulate cerebellothalamic and dentatorubral outflow. This strategy was further supported by our prior clinical experience demonstrating favorable outcomes with dual-lead DBS in patients with complex tremor phenotypes involving multiple motor networks.

Medical treatment provides objective improvement in only half of HT patients, especially in the resting component [3,6,7]. First-line medications include levodopa, trihexyphenidyl, and levetiracetam; while benzodiazepines, botulinum toxin injections, amantadine, and biperiden are second-line options [19]. Neurosurgical procedures such lesioning and DBS are occasionally employed in refractory cases. The VIM – the most caudal part of the ventrolateral thalamic nucleus [20] – is the most common interventional target for rare tremor syndromes, including HT, given its synchronously firing neuronal populations that act as tremorigenic pacemakers [21,22,23]. However, other regions have also been targeted in neurosurgical treatment of HT with success. A systematic review on HT treatment showed better results with DBS compared to medical therapy, with the GPi target superior to VIM in overall tremor reduction, especially in reducing rest tremor. The GPi target should especially be considered when other hyperkinetic movements (e.g., dystonia or chorea) are present. Increasing evidence suggests that single-target DBS may be insufficient for complex tremor syndromes such as HT, in which disruption of multiple motor control networks [24, 25]—including cerebello-thalamo-cortical and basal ganglia circuits—has been implicated. A recent systematic review of multiple-target DBS in non-parkinsonian movement disorders summarized the existing literature on dual- and multi-target approaches, including HT. Although limited primarily to case reports and small series, combined targeting strategies such as VIM + GPi, VIM + VOA/VOP, VIM + STN, and thalamic–subthalamic area combinations were generally associated with favorable tremor reduction and acceptable safety profiles [25]. These findings are further supported by recent individual reports and case series focusing specifically on combined thalamic and posterior subthalamic area targeting. Chong et al. described a patient with HT secondary to brainstem hemorrhage treated with a single trajectory traversing the VIM and PSA, resulting in marked and sustained tremor improvement over a 24-month follow-up without significant stimulation-related adverse effects, highlighting the feasibility of dual-target modulation along the same lead [26]. In a case series, Yilmaz et al. reported outcomes of bilateral VIM + PSA DBS in patients with rare tremor syndromes, including eight patients with HT [27]. Notably, stimulation was frequently delivered at ventral VIM and dorsal or ventral PSA contacts, supporting the concept that engaging adjacent nodal regions within the tremor network may enhance clinical efficacy when single-target stimulation is insufficient. Collectively, these observations support a network-based approach to DBS in HT and provide important context for the present case. We used two electrodes targeting the left GPi and the left VIM/PSA.

Although we included patient-reported improvements in day-to-day activities of daily living, task-based assessments (e.g., drawing or writing) may have provided additional insights into fine motor control and were not systematically captured in this case, representing a limitation. Additionally, validated scales to quantify dystonia and ataxia were not systematically applied, as the primary therapeutic focus and outcome of interest in this case was tremor improvement. Another limitation of this report is the lack of assessment of differential effects of the dual lead DBS on rest, postural, and intention tremor components, which was further complicated by the difficulty in distinguishing intention tremor from cerebellar appendicular ataxia in this patient.

This report highlights the potential of multi-target DBS and electrophysiological evaluation in managing complex, treatment-resistant hyperkinetic movement disorders.

## Additional File

The additional file for this article can be found as follows:

10.5334/tohm.1128.s1 Supplemental Figures. Figures 1 and 2.
