# Supplementary material for: Holmes Tremor in CADASIL Responsive to Multi-Targeting Deep Brain Stimulation (DBS): An Educational Case with Video and Electrophysiology
Source: Tremor Other Hyperkinet Mov (N Y). 2026 May 8;16:30. doi: 10.5334/tohm.1128 (PMC13155202; doi:10.5334/tohm.1128)
Supplement: Supplemental Figures. — Figures 1 and 2. [file tohm-16-1-1128-s1.pdf]

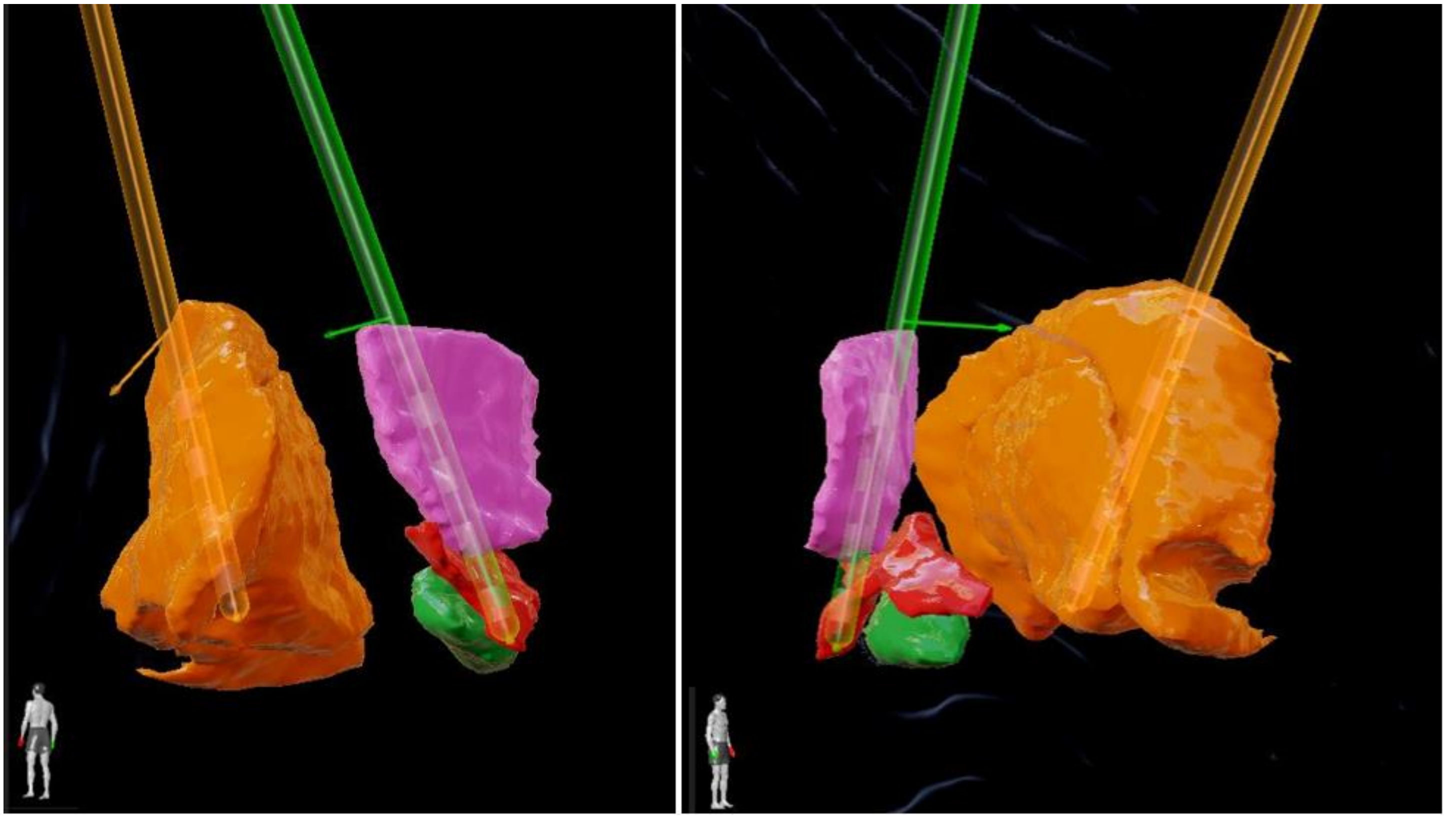

Supplemental Figure 1- 3D reconstruction of two left-sided DBS leads implanted with the Abbott Infinity™ system. The lateral lead targets the globus pallidus internus (orange), while the medial lead traverses the thalamic-subthalamic region**,** stimulating the ventral intermediate nucleus (purple) proximally and the posterior subthalamic area / zona incerta (red) distally. STN is depicted in green.

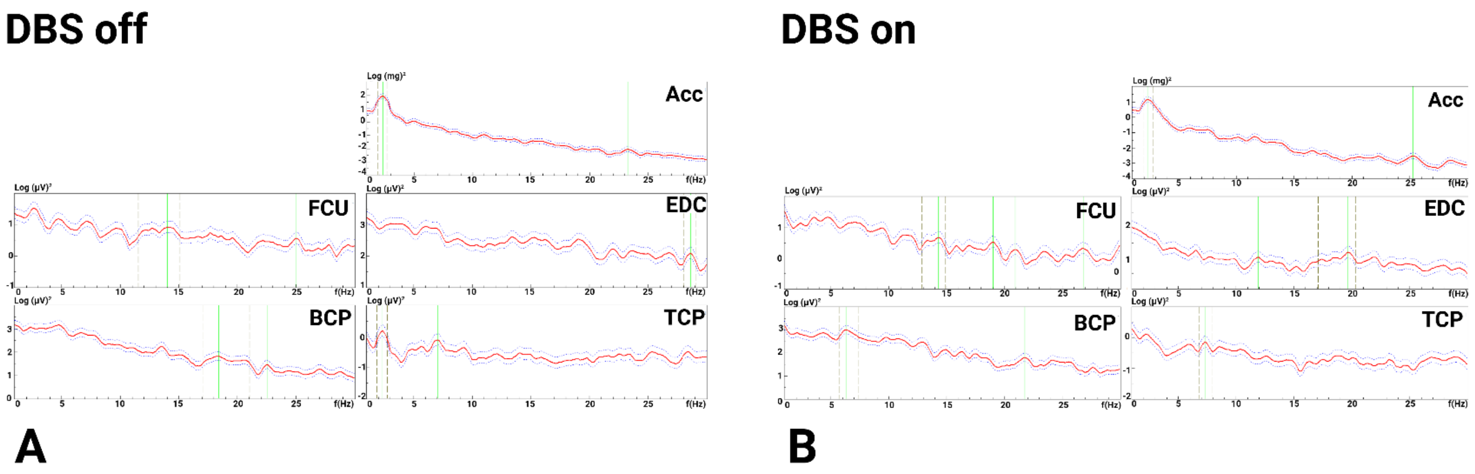

Supplemental Figure 2- Electromyography and accelerometry recording from the upper right limb in posture after deep brain stimulation surgery shows a 2.3-Hz tremor when stimulation is turned off, especially in the accelerometer, flexor carpi ulnaris, and triceps (A). With stimulation turned on, there is attenuation of the tremor, although a 2.3-Hz tremor is still observed in the accelerometer recording. Acc: accelerometer, BCP: biceps EDC: extensor digitorum communis; FCU: flexor carpi ulnaris; TCP: triceps
